# Creating Contacts Between Replication and Movement at Plasmodesmata – A Role for Membrane Contact Sites in Plant Virus Infections?

**DOI:** 10.3389/fpls.2020.00862

**Published:** 2020-07-03

**Authors:** Amit Levy, Jens Tilsner

**Affiliations:** ^1^Department of Plant Pathology, University of Florida, Gainesville, FL, United States; ^2^Citrus Research and Education Center, University of Florida, Lake Alfred, FL, United States; ^3^Biomedical Sciences Research Complex, The University of St. Andrews, St. Andrews, United Kingdom; ^4^Cell and Molecular Sciences, The James Hutton Institute, Dundee, United Kingdom

**Keywords:** plant virus, membrane contact site, replication, cell-to-cell movement, synaptotagmin, plasmodesmata

## Abstract

To infect their hosts and cause disease, plant viruses must replicate within cells and move throughout the plant both locally and systemically. RNA virus replication occurs on the surface of various cellular membranes, whose shape and composition become extensively modified in the process. Membrane contact sites (MCS) can mediate non-vesicular lipid-shuttling between different membranes and viruses co-opt components of these structures to make their membrane environment suitable for replication. Whereas animal viruses exit and enter cells when moving throughout their host, the rigid wall of plant cells obstructs this pathway and plant viruses therefore move between cells symplastically through plasmodesmata (PD). PD are membranous channels connecting nearly all plant cells and are now viewed to constitute a specialized type of endoplasmic reticulum (ER)-plasma membrane (PM) MCS themselves. Thus, both replication and movement of plant viruses rely on MCS. However, recent work also suggests that for some viruses, replication and movement are closely coupled at ER-PM MCS at the entrances of PD. Movement-coupled replication at PD may be distinct from the main bulk of replication and virus accumulation, which produces progeny virions for plant-to-plant transmission. Thus, MCS play a central role in plant virus infections, and may provide a link between two essential steps in the viral life cycle, replication and movement. Here, we provide an overview of plant virus-MCS interactions identified to date, and place these in the context of the connection between viral replication and cell-to-cell movement.

## Introduction: Plant Virus Movement in the Context of Infection

Viruses that infect plants have to overcome the barrier of the cell wall when moving between host cells. The only pathway available to them are plasmodesmata (PD) (Rojas et al., 2016; Pitzalis and Heinlein, 2017; Reagan and Burch-Smith, 2020), membranous channels traversing the wall, and plant viruses have evolved specialized transport systems consisting of virus-encoded movement proteins (MPs) to facilitate shuttling of infectious genomes through PD (Lucas, 2006; Tilsner et al., 2014; Heinlein, 2015; Hong and Ju, 2017). Viral cell-to-cell movement is a “race” against the antiviral mobile RNA silencing signal (Zhang et al., 2019) and usually occurs early in infection, often within a few hours after cell entry, when viral genomes are not yet abundant in the host cell (Derrick et al., 1992; Angell et al., 1996; Kawakami et al., 2004; Tilsner and Oparka, 2012). It is therefore crucial for the success of the infection that the movement system correctly selects viral genomes for transport. However, no characterized MPs so far have shown sequence specificity to their cognate viral genomes, and how transport specificity is achieved remains an important unresolved question (Tilsner and Oparka, 2012). Different plant viruses move either as encapsidated virions or as a non-virion, MP-containing ribonucleoprotein (RNP) complex, respectively (Lucas, 2006; Tilsner et al., 2014). In the former case, the specificity of transport depends on cognate interactions between MPs and capsid proteins (Nagano et al., 1997; Carvalho et al., 2003; Takeda et al., 2004), whilst specificity of encapsidation is achieved through capsid protein-nucleic acid secondary structure interactions (Rao, 2006) as well as through linking to (genome-specific) replication (Annamalai et al., 2008; Lee et al., 2011).

In the case of viruses moving as non-virion RNP, movement happens in competition with virion formation. As genome-specific replication proteins have also been implemented in cell-to-cell movement (Hirashima and Watanabe, 2003; Christensen et al., 2009) in addition to the non-sequence-specific MPs (Citovsky et al., 1990), transport specificity may be achieved through spatial co-compartmentation of the assembly of movement RNP complexes with replication. PD targeting of replication complexes may link directly with movement and the transported RNP could include the viral replicase or some of its components (Kawakami et al., 2004; Tilsner et al., 2013; Levy et al., 2015). Thus, in both virion- and non-virion RNP-transporting plant viruses, spatial coupling of replication to encapsidation/movement, or directly to movement is likely important to achieve specific transport of viral genomes. In agreement with their lack of sequence specificity, MPs can often complement transport of unrelated viruses (Latham and Wilson, 2008). However, this implies that there are probably few specific direct MP-replicase interactions involved in achieving movement specificity. Instead, coupling may be achieved through CP-replicase interactions (in virion-transporting viruses), localized translation of MPs at replication sites, joint MP-RNA and RNA-replicase interactions on the same genome (Tilsner and Oparka, 2012), or host factors. For instance in *Red clover necrotic mosaic virus*, the MP is recruited to replication complexes by the host protein, glyceraldehyde 3-phosphate dehydrogenase A (Kaido et al., 2014).

Recently, membrane contact sites (MCS) have emerged as host cell structures that are being exploited by plant viruses for both replication and movement, raising the intriguing possibility that they might also be involved in linking these processes and thus play crucial roles in plant virus infections. Here, we summarize the so far identified interactions of plant viruses with MCS, and discuss how they may contribute to linking replication and movement.

## Plant Membrane Contact Sites and Plasmodesmata

Whilst the presence of ER-PM contacts in plants has been known for some time (Hepler et al., 1990), it is only recently that the identities of the first ER-PM MCS proteins were revealed. These include the actin binding protein networked (NET) 3C, vesicle-associated membrane protein (VAMP)-associated protein 27 (VAP27), synaptotagmins (SYT) A/1, E/5 and 7 and multiple C2 domains and transmembrane region proteins (MCTPs) (Wang et al., 2014, 2016; Levy et al., 2015; Perez-Sancho et al., 2015; Brault et al., 2019). Reticulons, some of which bind SYTA/1 and VAP, and localize to PD, were shown to be MCS proteins in non-plant systems, and may represent another component of plant MCSs (Kriechbaumer et al., 2015; Caldieri et al., 2017). VAPs are ER integral membrane proteins that interact with various lipid binding/sensing/transport proteins, including the oxysterol-binding proteins (OSBPs) (Lev et al., 2008). VAPs and OSBP-related proteins (ORPs) function in lipid transfer, and were shown to localize to ER-PM contact sites in yeasts (Schulz et al., 2009; Stefan et al., 2011; Manford et al., 2012; Siao et al., 2016).

Mammalian extended-synaptotagmins (E-SYTs) localize at ER-PM junctions, and take part in tethering the ER to the PM (Giordano et al., 2013). Like classical SYTs, E-SYTs contain an N-terminal, ER-inserted transmembrane domain, and several C-terminal C2 domains, which can bind PM lipids. However, they also possess an additional central domain called the synaptotagmin-like mitochondrial and lipid binding protein (SMP) domain (Giordano et al., 2013). SMP domains are lipid-binding modules that are proposed to have a specialized role in lipid transfer at MCS (Schauder et al., 2014; Yu et al., 2016), and are necessary to localize E-SYTs to ER-PM MCSs (Toulmay and Prinz, 2012). Arabidopsis SYTs contain the SMP domain, and localize to ER-PM sites where they act as membrane tethers (Levy et al., 2015; Perez-Sancho et al., 2015; Siao et al., 2016; Ishikawa et al., 2018, 2020). Like E- SYTs and plant SYTs, MCTPs bind to the PM with C2 domains and are ER-anchored by a transmembrane region, but their C2 domains are located at the N-, and the transmembrane region, which spans the membrane multiple times, at the C-terminus (Brault et al., 2019). MCTPs do not contain an SMP domain.

Most of the plant ER is situated close to the PM (referred to as “cortical ER”), and is expected to be strongly anchored to the PM (Sparkes et al., 2011; Chen et al., 2012). The Arabidopsis genome contains at least 10 genes encoding for proteins with an SMP domain, including all Arabidopsis SYTs (Levy et al., 2015), double the number of *Homo sapiens* (Lee and Hong, 2006), suggesting that MCS may play a uniquely central role in plant cell signaling, compared to that in mammalians cells. About a third of SYTA/1-labeled contact sites are localized adjacent to PD (Schapire et al., 2008; Levy et al., 2015). Whilst plant SYTs appear to be mainly localized at PD entrances, several members of the MCTP family have now been found to be highly enriched in purified PD fractions and localize predominantly to the inside of the channels, making them likely candidates for connecting the ER and PM inside PD (Liu et al., 2012; Vaddepalli et al., 2014; Brault et al., 2019). Several MCTP family proteins have been implicated in macromolecular trafficking through PD. For instance, FT-INTERACTING PROTEIN 1 (FTIP1)/MCTP1 is required for systemic transport of the florigen signal flowering locus T (FT) through the phloem (Liu et al., 2012), QUIRKY (QKY)/MCTP15 promotes non-cell-autonomous signaling by the receptor-like kinase STRUBBELIG (SUB) (Vaddepalli et al., 2014) and MCTP3/4 negatively affect movement of SHOOTMERISTEMLESS (STM), a class I KNOTTED1 (KN1)-like homeobox (KNOX) protein, in the shoot apical meristem (Liu et al., 2018), whereas MCTP3/4 knock out reduces GFP movement in leaves (Brault et al., 2019). These results indicate a tight connection between MCSs and intercellular communication in plants (Tilsner et al., 2016).

## MCS and Viral Replication

The majority of RNA viruses have predominantly cytoplasmic infection cycles and replicate on the surface of various cellular membranes. In the process of establishing their membrane-bound replication complexes, they extensively modify the membrane architecture into novel structures like invaginated spherules or tubules, or stacked membranes, known as viral replication complexes (VRCs), viroplasms or virus factories (Miller and Krijnse-Locker, 2008; den Boon et al., 2010; Xu and Nagy, 2014). These structures may provide a variety of functions in infection: (1) hide viral replication intermediates such as double-stranded RNA from cellular defense surveillance (Överby et al., 2010), (2) provide a scaffold for replication complexes (Gouttenoire et al., 2014) and activate replication enzymes (Xu and Nagy, 2017), (3) compartmentalize metabolic energy delivery (Lin et al., 2019), translation (Bamunusinghe et al., 2009; Mäkinen and Hafren, 2014), and virion assembly (Annamalai and Rao, 2006), and (4) provide access to cellular membrane trafficking routes. VRC formation often involves *de novo* lipid synthesis, and shuttling of suitable lipids to the replication site. In turn, inhibition of lipid production can cause VRC disassembly and inhibit replication (Bamunusinghe et al., 2009; Lyn et al., 2009). Given the emerging role of MCS in non-vesicular lipid shuttling, it is perhaps not surprising that viruses have been found to hijack MCS components to establish their “factories.”

SYTA/1 was shown to play a role in the formation of VRCs during *Turnip vein clearing virus* (TVCV; Tobamovirus) infection - in a *syta* mutant TVCV VRCs are significantly smaller than in wild-type plants (Levy et al., 2015). Although dispensable for replication, the MP of the closely related *Tobacco mosaic virus* (TMV; Tobamovirus) plays a role in the formation of replication sites (Mas and Beachy, 1999), which could be related to its interaction with SYTA/1 (Lewis and Lazarowitz, 2010). SYTA/1 accumulates inside the TVCV VRCs, and, through its putative lipid transfer properties (Yu et al., 2016) may support the formation of the VRC by inducing the redistribution of lipids, similar to VAP/OSBPs in other viruses (see below). Reticulons, ER-tubulating proteins which promote formation of ER-PM and ER-mitochondrial MCS in animals (Caldieri et al., 2017), and which bind Arabidopsis SYTA/1 (Kriechbaumer et al., 2015) and may therefore be components of ER-PM MCSs, associate with *Brome mosaic virus* (BMV; Bromovirus) 1a replicase component and play a role in VRC formation, likely by stabilizing positive membrane curvature at the openings of ER-derived spherules containing the replication complexes (Diaz et al., 2010).

The role of *Tomato bushy stunt virus* (TBSV; Tombusvirus) p33 in VRC formation was studied in detail. The tombusvirus p33 replication protein interacts with an ER-resident VAP protein (Scs2 in yeast) and with several OSBP homologs (called OSBP-related proteins or ORPs in plants and yeast) to form MCSs between the ER and peroxisomes (the site of TBSV replication). The recruited ORPs mediate the transfer of sterols to peroxisome membranes, resulting in the enrichment of sterol in the replication organelles (Barajas et al., 2014). VAP27 proteins were also shown to interact with the *Cowpea mosaic virus* (CPMV; Comovirus) 60K protein, required for the formation of replicative vesicles of the virus (Carette et al., 2002), which includes *de novo* lipid synthesis (Carette et al., 2000). Thus, manipulation of lipid transfer at MCS could represent one general mechanism for the formation of virus replication bodies, and these processes seem to have similarities in both plant and non-plant systems (Amarilio et al., 2005; Lev et al., 2008; Manford et al., 2012; van der Schaar et al., 2016; Galindo et al., 2019).

## MCS and Viral Movement

**FIGURE 1:**
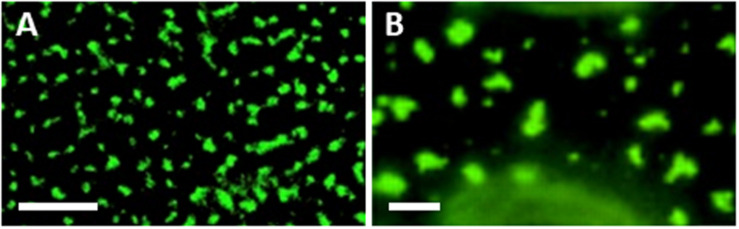
Similarity between localizations of ER-PM MCS and movement proteins. **(A)** SYTA/1-GFP transient expression in *Nicotiana benthamiana* leaf epidermal cells. Adapted from Levy et al. (2015) (Figure 1A), used with permission. **(B)** *N. benthamiana* leaf epidermal cell infected with Tobacco mosaic virus expressing MP-GFP. Adapted from Heinlein et al. (1998) (Figure 2J), used with permission. Scale bars, 5 μm.

Targeting MCS, and especially ER-PM MCSs that are localized adjacent to PD, could provide an ideal location for viruses to simultaneously achieve both membrane remodeling toward VRC formation and PD targeting. The association between viral MPs and SYTA/1 was studied in detail. SYTA/1 was found to be required for the movement of viruses from the Tobamovirus [TMV, TVCV, and *Youcai mosaic virus* (YoMV)], Potyvirus [*Turnip mosaic virus* (TuMV)] and Begomovirus [*Cabbage leaf curl virus* (CaLCuV)] genera (Uchiyama et al., 2014; Cabanillas et al., 2018; Ishikawa et al., 2020). SYTA/1 forms a complex with SYTE/5 and SYT7 that likely also involves actin (Ishikawa et al., 2020), and was shown to localize at PD entrances (Levy et al., 2015; Ishikawa et al., 2020). SYTA/1 interacts with the movement proteins of TMV, TVCV, CaLCuV and *Squash leaf curl virus* (SqLCV; Begomovirus) (Lewis and Lazarowitz, 2010; Levy et al., 2015). SYTA/1 was required for the targeting of MP^TVCV^ and MP^TMV^ to PD (Levy et al., 2015; Yuan et al., 2018), and the SYTA/1-E/5-7 complex for the cell-to-cell movement of MP^YoMV^ (Ishikawa et al., 2020). It was also shown that the PD localization signal of MP^TMV^ interacts with SYTA/1 both *in vitro* and *in vivo* (Yuan et al., 2018). The *Fig mosaic virus* (FMV; Emaravirus) MP also localizes to ER-PM MCSs, and this localization was required for PD targeting of MP^FMV^ (Ishikawa et al., 2017). This list demonstrates the wide diversity of plant viruses that associate with the ER-PM MCS, and utilize SYT proteins, including viruses with different genomes (DNA/RNA), and different movement strategies. Intriguingly, what is now recognized as the typical localization pattern of ER-PM MCS proteins (peripheral puncta which are found on all sides of the cell including the upper surface of epidermal cells where there are no PD, and which can be irregular in shape or extend along peripheral ER tubules) appears remarkably similar to the localizations of viral movement proteins when overexpressed or expressed in PD-less protoplasts (Heinlein et al., 1998) (Figure 1). This suggests that MPs may associate with ER-PM MCS to achieve PD targeting at a sub-set of these structures. Whilst Tobamovirus MPs require SYTA/1 for PD targeting (Levy et al., 2015; Yuan et al., 2018), SYTA/1 had no effect on GFP secretion or PD targeting of PD LOCATED PROTEIN 1 (PDLP1), which reaches PD through the secretory pathway (Thomas et al., 2008; Levy et al., 2015), indicating that Tobamovirus MPs use an alternative pathway to the PD. Similarly, knock out or overexpression of truncated forms of the ER-PM MCS localized SYTs A/1 and E/5 inhibited TuMV movement, whereas knock out/overexpression of truncations of the Golgi-associated SYTS B/2 and F/6, or inhibition of ER-Golgi transport, actually enhanced movement (Cabanillas et al., 2018). Other viruses which do not require the secretory pathway for PD targeting and/or movement include CPMV (Pouwels et al., 2002) and *Potato virus X* [PVX; Potexvirus (Schepetilnikov et al., 2005)]. Collectively, these results suggest that MCSs may play an important role in “unconventional” PD targeting that does not involve the secretory pathway.

## Conclusion: Co-Replicational Movement

The integration and coordination of cell-to-cell transport with other parts of the infection process as described above require that the activities of movement proteins are spatially and temporally linked to replication, encapsidation and suppression of host defenses. One way in which a regulated distribution of viral progeny between movement and other infection processes, and ordered assembly and modification of RNP complexes could occur is through co-compartmentalization. For several viruses, there is now strong evidence that replication and movement indeed become spatially coupled, and furthermore, that at least in some cases this actually happens at PD. In TMV, viral replicase, which is directly implicated in movement, is found in MP-organized modified ER-membranes at the entrances of PD (Saito et al., 1987; Szecsi et al., 1999; Kawakami et al., 2004), and similar structures are observed in the closely related TVCV (Levy et al., 2015). In TuMV, replicative vesicles formed by p6K2 protein, the membrane anchor for the potyviral replicase, are also recruited to PD by the CI and P3N-PIPO MPs (Schaad et al., 1997; Movahed et al., 2017; Chai et al., 2020). In PVX, MPs organize the structure of VRCs (Tilsner et al., 2012), and some of these replication sites are found at PD openings. Additionally, in this case, virions accumulate all around cytoplasmic VRCs, whereas at PD-anchored replication sites, CP is only found inside the channels, raising the possibility that PVX movement is co-replicational, i.e., all nascent progeny virus might be inserted directly into PD as it emerges from the viral replicase (Tilsner et al., 2013). PD-localized replication has also been proposed for a plant DNA virus (Rodriguez et al., 2014; Schoelz et al., 2016).

**FIGURE 2:**
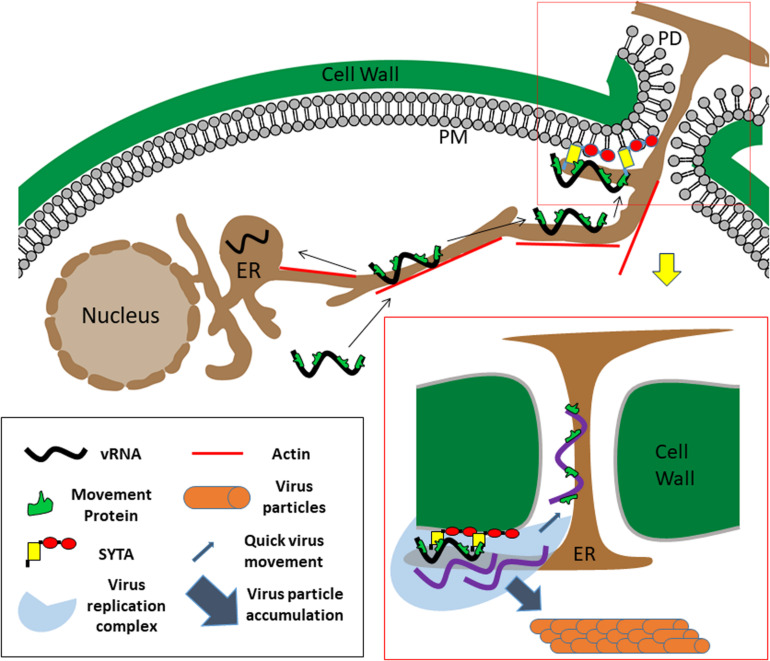
Model for Tobamovirus movement. Viral ribonucleoprotein complexes containing the viral RNA (black), movement protein, and likely the replicase (not shown) enter the newly infected cell and slide along the ER endomembrane, until reaching and attaching to SYTA/1 ER-PM anchors, some of them located adjacent to PD. Utilizing MCS components, the virus will modify the ER membranes to form a replication complex (shown in boxed area on the bottom right). From PD-anchored replication complexes, new virus nucleoprotein complexes can exit directly into PD and quickly move on the plasmodesmal ER membrane to the next cell. MCS may also play an additional role in regulating PD aperture. Additional replication of viral RNA (purple) (as well as replication at complexes not localized at PD) will lead to accumulation of viral progeny in the cell in the form of virions. vRNA: viral RNA. Elements in this figure are not to scale.

MCS play important roles in both cell-to-cell movement and replication of plant viruses, and may link these processes. A model for Tobamovirus movement and replication is shown in Figure 2. We suggest SYTs at PD entrances serve as a docking point for Tobamovirus MP-containing RNPs or VRCs gliding along the ER. As only a portion of the MCS are situated next to PD, this model supports the notion that virus movement is a limited process compared to the subsequent replication (Tilsner and Oparka, 2012). MP interaction with ER-PM tethers, particularly MCTPs, could also have a role in the ability of MPs to increase the PD size exclusion limit, required for movement. ER-PM tethers may not only determine the diameter of the cytoplasmic compartment of PD between the PM and the ER, but also contribute to the correct localization of PD regulating proteins (Brault et al., 2019), both directly through protein-protein interactions, and indirectly by contributing to PD membrane lipid homeostasis (Tilsner et al., 2016). Similarly, reticulons at PD may play a role in regulating PD aperture directly via maintaining constriction of the plasmodesmal ER (Tilsner et al., 2011; Knox et al., 2015) or indirectly by recruitment of other MCS components. Thus, MP interactions with MCSs may be important for maintaining PD in an open state. New reports also connect MCSs to additional cellular processes that likely have a role in virus replication and movement such as reactive oxygen species (ROS) signaling, calcium signaling, autophagy and organelle trafficking and positioning (Prinz et al., 2020). These diverse functions of MCSs are likely to affect virus infections as well. For example, opening of PD by MPs may be related to altering MCS Ca^2+^ and ROS signaling adjacent to PD, thereby regulating turnover of the polysaccharide callose, whose accumulation in the cell wall around PD entrances constricts their opening and reduces cell-to-cell transport (Amsbury et al., 2017). MCS regulation may also affect autophagy, another cellular process with an emerging role in plant virus movement (Clavel et al., 2017). Thus, MCSs may not only connect membranous cellular organelles, but also the different sub-processes of viral infections. Further developments in the field of plant MCS research will be of major interest to plant virologists, and in turn, the way in which plant viruses exploit MCSs in their host cells will help to elucidate the functions of these structures.

## Author Contributions

AL and JT wrote and edited the manuscript. Both authors contributed to the article and approved the submitted version.

## Conflict of Interest

The authors declare that the research was conducted in the absence of any commercial or financial relationships that could be construed as a potential conflict of interest.

## Abbreviations

ER: endoplasmics reticulum
MCS: membrane contact site
MP: movement protein
PD: plasmodesmata
PM: plasma membrane
RNP: ribonucleoprotein
VRC: viral replication complex.
